# Supplementary material for: Slow-light-enhanced energy efficiency for graphene microheaters on silicon photonic crystal waveguides
Source: Nat Commun. 2017 Feb 9;8:14411. doi: 10.1038/ncomms14411 (PMC5309776; doi:10.1038/ncomms14411)
Supplement: Supplementary Information — Supplementary Figures and Supplementary Notes [file ncomms14411-s1.pdf]

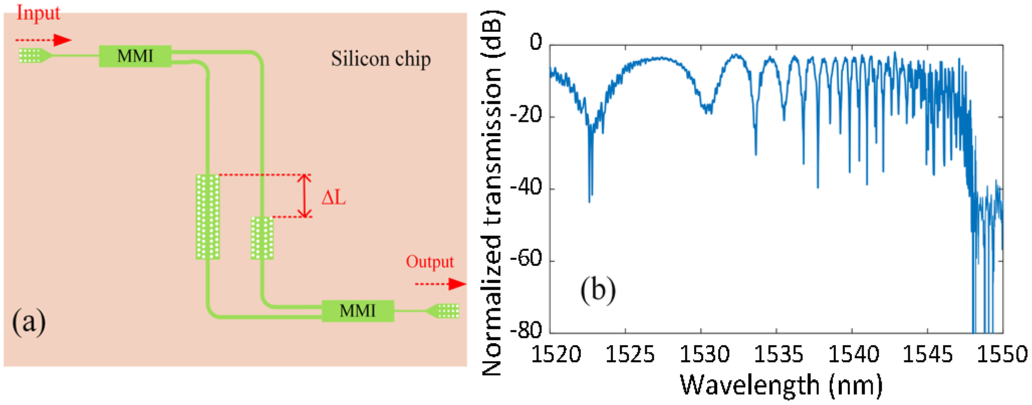

Supplementary Figure 1: Group index measurement. (a) Schematic of the group index measurement device, which consists of a Mach–Zehnder interferometer with photonic crystal waveguide incorporated in each arm. The two photonic crystal waveguides in the two arms have length difference ΔL in order to introduce comb-like interference transmission for the Mach–Zehnder interferometer, through which group index of the photonic crystal waveguide can be derived. (b) Measured transmission spectrum.

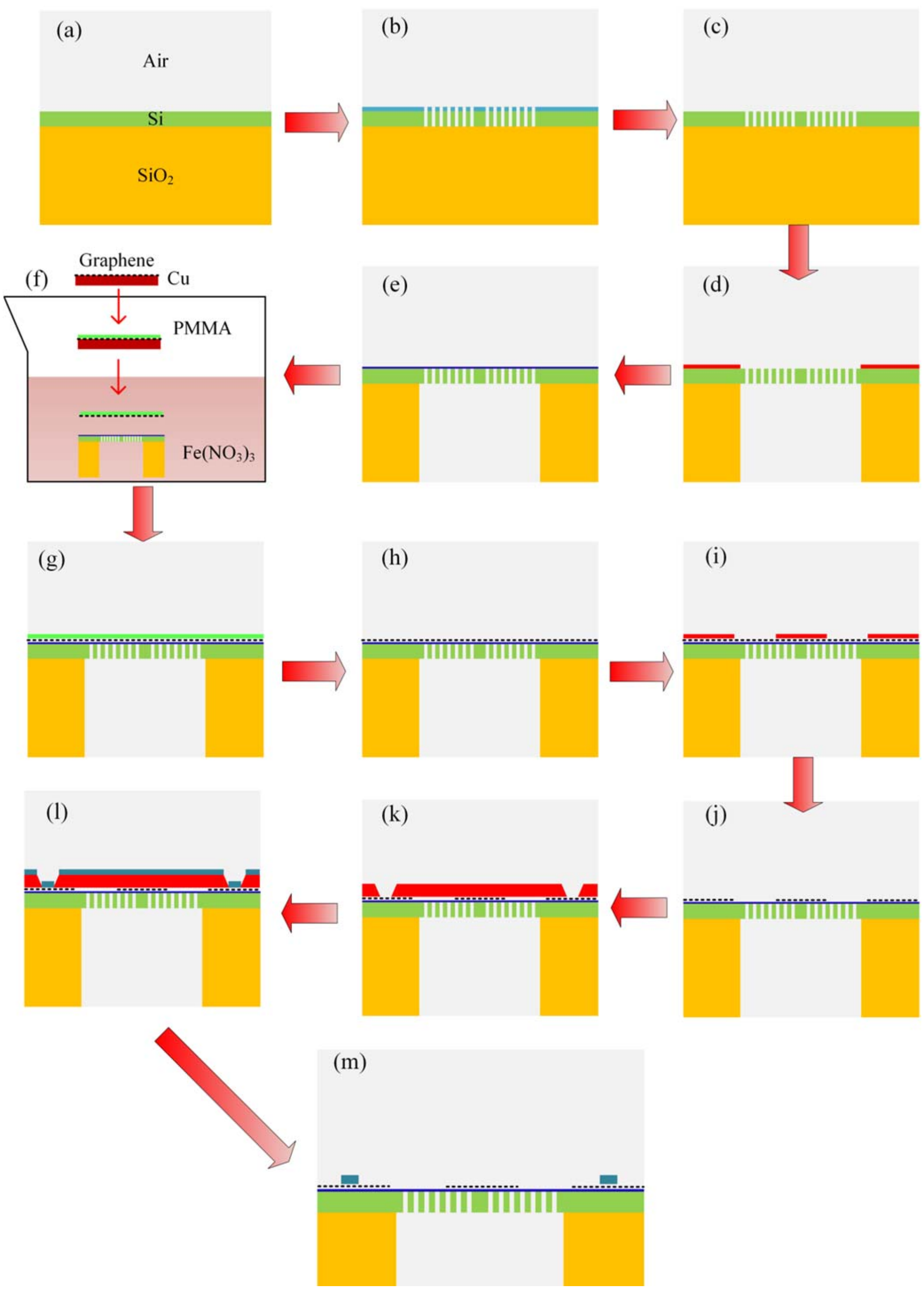

Supplementary Figure 2: Detailed process flow of the silicon photonic crystal with graphene heater. (a)-(c): E-beam lithography and ICP etch for device fabrication on silicon layer. (d) Photonic crystal waveguide membranization. (e) Al2O3 layer deposition. (f)-(h): Graphene wet transfer. (i)-(j): Graphene heater definition. (k)-(m): Metal contacts fabrication.

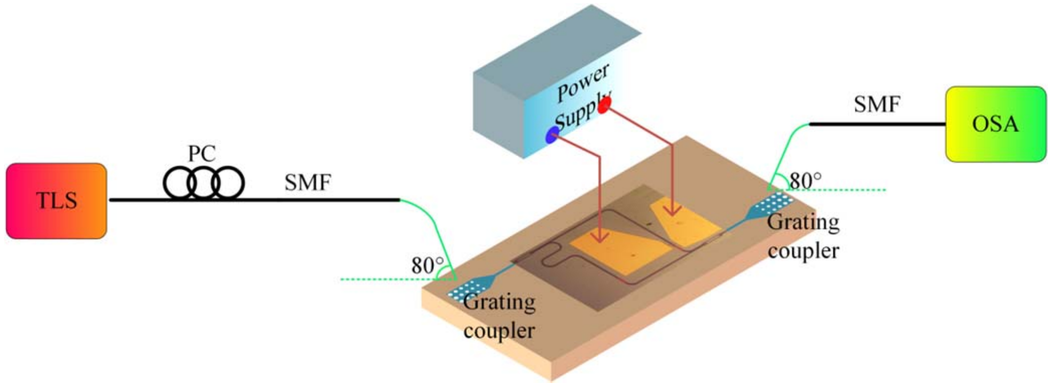

Supplementary Figure 3: The experimental setup. The input light from a tunable laser source is polarization controlled by a polarization controller. The light on the TE polarization state is injected into the chip through grating coupler with coupling angle of 80 degrees. The output spectral response is recorded by an optical spectrum analyzer. A static electrical signal is applied to the chip using a power supply. When dynamic response was measured, the power supply is replaced by a waveform generator, and the modulated signal is received by a photodetector and recorded by an oscilloscope.

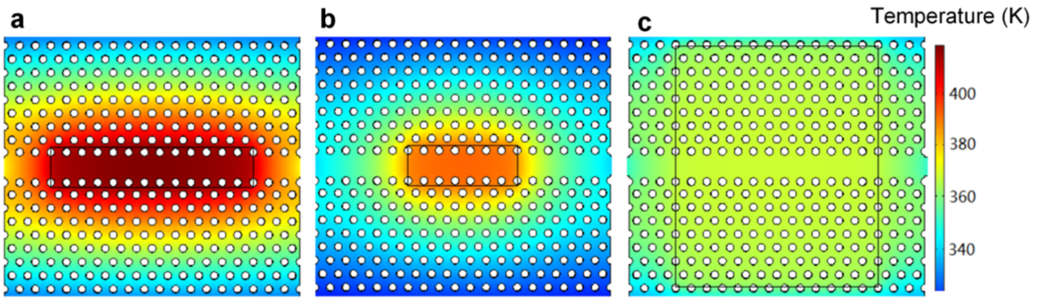

Supplementary Figure 4: Temperature distribution of different structures with the same normalized power. Graphene heater with the length of (a) 5 μm and (b) 2.5 μm for the Z-shaped design. Here, only straight part heating source of the Z-shaped graphene heater is considered. (c) Straight-shaped graphene heater with the length of 5 μm.

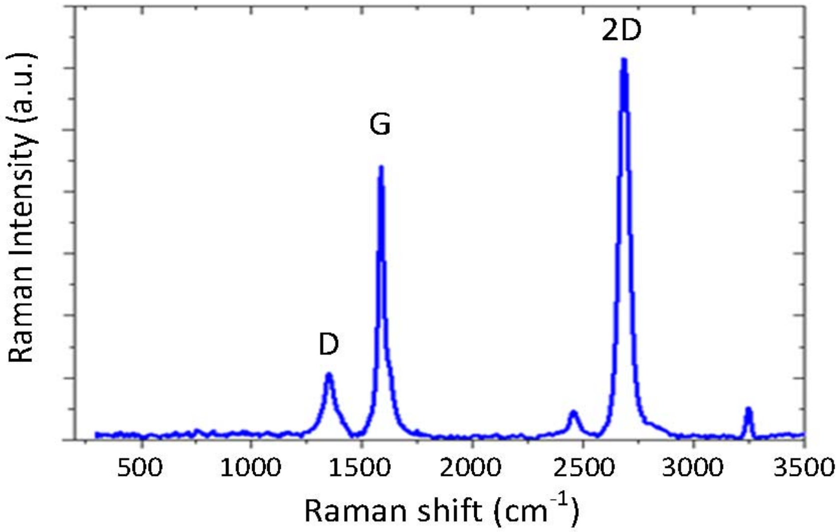

Supplementary Figure 5: Raman spectrum of graphene after the wet-transfer process. A prominent D peak appears at 1358 cm^{-1}. The observation suggests the presence of moderate defects during the wet-transfer process.

Supplementary Note 1: Group index measurements.

To characterize the group index of the photonic crystal waveguide, we fabricated a MZI structure consisting of two photonic crystal waveguides with different lengths, as shown in Supplementary Figure 1(a). The length difference ΔL is set to 100 μm. The measured transmission spectrum of the device is depicted in Supplementary Figure 1(b). By extracting the free spectra range (FSR) of the device, the group index of photonic crystal waveguide $n_{\mathrm{g\_PCW}}$ can be obtained through:

$$n_{\mathrm{g\_PCW}} = \frac{c}{\Delta L * FSR} + n_{\mathrm{g\_Si}} \quad \text{(Supplementary Equation 1)}$$

where c is the speed of light in vacuum, $n_{\mathrm{g\_Si}}$ is the group index of silicon strip waveguide, which is calculated to be 4.3.

Supplementary Note 2: Fabrication process.

The details of the fabrication process can be found in Supplementary Figure 2. The silicon photonic crystal waveguide is fabricated using e-beam lithography (JEOL JBX-9300FS and e-beam resist ZEP520A) and inductively coupled plasma (ICP) etching (STS Advanced Silicon Etcher), shown in Supplementary Figure 2(a)-Supplementary Figure 2(c). Then standard ultraviolet (UV) lithography (photoresist: AZ5214E) is performed to define region where membranization is required. Buffered hydrofluoric acid (BHF) is used to etch the SiO_2 buried layer below the photonic crystal waveguides (Supplementary Figure 2 (d)). An 11nm Al_2O_3 layer is deposited on the wafer using atomic layer deposition (Picosun ALD model R200), shown in Supplementary Figure 2 (e), in order to avoid the direct contact between metal contacts and the silicon waveguide as well as to improve the surface smoothness. AZ resist is spin-coated onto the graphene covered copper foil and dried at 170 °C for 1 min. By etching away the copper foil in a $Fe(NO_3)_3/H_2O$ solution, AZ/graphene membrane is then obtained and transferred onto the silicon slab. Finally, the AZ resist is dissolved in acetone and graphene wet transfer is finished (Supplementary Figure 2 (f)- Supplementary Figure 2 (h)). Afterwards, standard UV lithography and oxygen plasma etching are performed to define the shape of the graphene heater

(Supplementary Figure 2 (i)- Supplementary Figure 2 (j)). At last, the metal contacts are formed by another standard UV lithography, metal deposition and lift-off process (Supplementary Figure 2 (k)- Supplementary Figure 2 (m)).

Supplementary Note 3: Experimental setup.

The experimental setup for characterizing the device is shown in Supplementary Figure 3. The input light is emitted from a tunable laser source (TLS, ANDO AQ4321A). A polarization controller (PC) is used to select the TE polarization state of the input light before it is injected into the chip through the grating coupler. The output light is coupled into the single mode fiber (SMF) using another grating coupler. The spectral response is recorded by an optical spectrum analyzer (OSA, AQ6317B). A static electrical signal is applied to the chip using a power supply (Keithley 238, High Current Source Measure Unit). When we measure the dynamic response, the power supply is replaced by a waveform generator (Stanford Researcher Systems Inc., Model DG 535, Four Channel Digital Delay/Pulse Generator). The modulated signal is received by a photodetector, which converts the optical signal to the electrical signal, which is then recorded by an oscilloscope.

Supplementary Note 4: Thermal distribution calculation.

To study the temperature distributions for different structures, we have calculated the temperature distributions with respect to different length and shape of the graphene layer as follows from the same heating power. By solving the Poisson equation, we developed a 3D numerical model to achieve both temperature distribution and dynamic response. In order to see the shape effect clearly, we only considered the contributions from the central part when having the same normalized heat power. As seen from Supplementary Figure 4(a) and Supplementary Figure 4(b), the graphene-PhCW with longer length can have a larger temperature increase under the same normalized heating power. Besides, according to Supplementary Figure 4(a) and Supplementary Figure 4(c), the full coverage graphene heater on the photonic crystal waveguide, i.e. Straight-shaped (Supplementary Figure 4(c)), results in a much

lower temperature increase compared to the structure covering the central part of photonic crystal waveguide (Supplementary Figure 4(a)). Therefore, the tuning efficiency of the narrow-width long-length graphene heater can provide high heating efficiency under the same normalized power.

Supplementary Note 5: Raman analysis of graphene.

Here, we use Raman spectroscopy to examine the quality of graphene after the wet-transfer process. For the pristine CVD graphene, normally there is only a very small Raman D peak, indicative of the good structural quality of graphene. Supplementary Figure 5 shows the Raman spectrum of graphene after the wet-transfer process, where we observe a prominent D peak appearing at 1358 cm^{-1}. The observation suggests the presence of defects during the wet-transfer process.

Supplementary Note 6: Theoretical calculation.

To achieve the theoretical model of the slow light enhanced efficiency, we employ the perturbation theory. As the phase shift $\Delta\varphi$ can be expressed as below, where Δk is the wavevector shift, L is the length of the PhCW.

$$\Delta\varphi = \Delta k * L \quad \text{(Supplementary Equation 2)}$$

According to the perturbation theory in photonic crystal waveguide, we could obtain that:

$$\Delta k = \left(\frac{\partial\omega}{\partial k}\right)^{-1} * \Delta\omega \quad \text{(Supplementary Equation 3)}$$

$$\Delta\omega = -\frac{\omega}{2}\frac{\langle E|\Delta\varepsilon|E\rangle}{\langle E|\varepsilon|E\rangle} \quad \text{(Supplementary Equation 4)}$$

Therefore, we could obtain that

$$\Delta k = \frac{\omega}{2} * \frac{\Delta\varepsilon}{n_{\mathrm{Si}}^2} * \frac{f}{c} * n_{\mathrm{g}} \quad \text{(Supplementary Equation 5)}$$

Note that

$$\Delta\varepsilon \approx 2 * \Delta n * n_{\mathrm{Si}} \quad \text{(Supplementary Equation 6)}$$

Substitute Supplementary Equations (3-6) to Supplementary Equation (1), then we could obtain that,

$$\Delta\varphi = \left(\frac{\omega}{c}\right) * a * \Delta T * f * L * \left(\frac{n_{\mathrm{g}}}{n_{\mathrm{Si}}}\right) \quad \text{(Supplementary Equation 7)}$$

Where a is the thermo-optic coefficient, $f \equiv \frac{\langle E|\varepsilon|E\rangle_{\mathrm{d}}}{\langle E|\varepsilon|E\rangle_{\mathrm{a}}}$ is the filling fraction defining the fraction of the optical field in silicon.

To obtain the relationship between phase shift and the resonance dip, resonant condition of the MZI is employed:

$$\frac{2\pi}{\lambda_0} * n_{\mathrm{Si}} * \Delta L = (2k+1)\pi \qquad \text{(Supplementary Equation 8)}$$

Where λ_0 is the resonance wavelength before heating, n_{Si} is the effective index of the silicon waveguide, ΔL is the length difference between the two arms of the MZI and k is a positive integer. When the phase shift is induced to the one arm of the MZI, we can achieve that,

$$\frac{2\pi}{\lambda_1} * n_{\mathrm{Si}} * \Delta L + \Delta\varphi = (2k+1)\pi \qquad \text{(Supplementary Equation 9)}$$

Note that,

$$\Delta\varphi = \left(\frac{2\pi}{\lambda_1}\right) * a * \Delta T * f * L * \left(\frac{n_{\mathrm{g}}}{n_{\mathrm{Si}}}\right) \qquad \text{(Supplementary Equation 10)}$$

$$\lambda_0^2 \approx \lambda_0 * \lambda_1 \qquad \text{(Supplementary Equation 11)}$$

Where λ_1 is the resonance wavelength after heating, n_{g} is the group index of the PhCW and ΔL is the length difference between the two arms of the MZI.
Combining the Supplementary Equations (8-11), then we can achieve that:

$$\Delta\lambda = \frac{\Delta\varphi\lambda_0^2}{2\pi n_{\mathrm{Si}}\Delta L} \qquad \text{(Supplementary Equation 12)}$$
